# Treatment of polymyalgia rheumatica: British Society for Rheumatology guideline scope

**DOI:** 10.1093/rap/rkae002

**Published:** 2024-02-14

**Authors:** Task Toyoda, Zoe Armitstead, Sampada Bhide, Serge Engamba, Emma Henderson, Claire Jones, Pieter MacKeith, Janice Maddock, Gary Reynolds, Nicola Scrafton, Manil Subesinghe, Sujith Subesinghe, Helen Twohig, Sarah L Mackie, Max Yates

**Affiliations:** Leeds Institute of Rheumatic and Musculoskeletal Medicine, University of Leeds, Leeds, UK; NIHR Leeds Biomedical Research Centre, Leeds Teaching Hospitals NHS Trust, Leeds, UK; Department of Rheumatology, Leeds Teaching Hospitals NHS Trust, Leeds, UK; East Elmbridge Primary Care Network, Kingston Hospital NHS Foundation Trust, Kingston upon Thames, UK; Norwich Practices Health Centre, Norwich, UK; Department of Rheumatology, Stockport NHS Foundation Trust, Stockport, UK; Powys Teaching Health Board, Bronllys Hospital, Brecon, UK; Norwich Medical School, University of East Anglia, Norwich, UK; Patient Expert, PMRGCAuk, UK; Centre for Immunology and Inflammatory Diseases, Massachusetts General Hospital, Boston, USA; Department of Rheumatology, Northumbria Healthcare NHS Foundation Trust, Newcastle upon Tyne, UK; King’s College London and Guy’s and St Thomas’ PET Centre, London, UK; Department of Cancer Imaging, School of Biomedical Engineering and Imaging Sciences, King’s College London, London, UK; Department of Rheumatology, Guy’s and St Thomas’ NHS Foundation Trust, London, UK; School of Medicine, Keele University, Keele, UK; Leeds Institute of Rheumatic and Musculoskeletal Medicine, University of Leeds, Leeds, UK; NIHR Leeds Biomedical Research Centre, Leeds Teaching Hospitals NHS Trust, Leeds, UK; Norwich Medical School, University of East Anglia, Norwich, UK; Rheumatology Department, Norfolk and Norwich University Hospitals NHS Foundation Trust, Norwich, UK

**Keywords:** polymyalgia rheumatica, management, treatment

## Abstract

The last British Society for Rheumatology (BSR) guideline on PMR was published in 2009. The guideline needs to be updated to provide a summary of the current evidence for pharmacological and non-pharmacological management of adults with PMR. This guideline is aimed at healthcare professionals in the UK who directly care for people with PMR, including general practitioners, rheumatologists, nurses, physiotherapists, occupational therapists, pharmacists, psychologists and other health professionals. It will also be relevant to people living with PMR and organisations that support them in the public and third sector, including charities and informal patient support groups. This guideline will be developed using the methods and processes outlined in the BSR Guidelines Protocol. Here we provide a brief summary of the scope of the guideline update in development.

The guideline will be developed using the methods and processes outlined in Creating Clinical Guidelines: Our Protocol [1].

## Why the guideline is needed

The current BSR guideline for PMR was published in 2009 [2]. In 2015, treatment recommendations for PMR were produced collaboratively by EULAR and ACR in 2015 [3]. Since then, new PMR clinical trial evidence has emerged and a major guideline update is needed.

The average age of diagnosis of PMR is 72 years and it is rarely diagnosed in those <50 years of age [4]. In the UK, PMR is primarily managed in primary care, with specialist referral for selected cases. However, there is a need to design better care pathways for PMR [5] that reflect the aspiration of the National Health Service (NHS) long-term plan to deliver more personalized therapeutic options and person-centred care [6]. Redesign of care pathways for PMR will need updated evidence-based treatment recommendations to determine optimal care for these patients.

In this guideline we will seek to identify evidence relating to treatment of patients with PMR; we will not cover methods of PMR diagnosis. Clinical diagnosis is a matter for the judgement of an appropriately trained and experienced clinician, supported by targeted investigations depending on the clinical presentation of the individual patient and the context and setting of care. We will not cover immune checkpoint inhibitor–associated PMR because this is a special case in which the context of treatment must take into account the imperative for treatment of the underlying neoplastic condition for which the immune checkpoint inhibitor is being given.

## Key facts and figures

PMR is an inflammatory rheumatic disease characterized by musculoskeletal stiffness and pain in a proximal distribution, commonly affecting those >50 years of age. The estimated incidence of PMR in the UK is ≈96/100 000 per year over the age of 40 years; epidemiological studies of PMR from Northern European countries have generally reported a higher incidence and prevalence than studies from other countries [7, 8]. PMR affects ≈1% of the UK population, with a predisposition towards women (lifetime prevalence of 2.4% in women *vs* 1.7% in men) and incidence increases with age [8, 9]. There is no conclusive evidence on the aetiology of PMR, although a combination of genetic factors and environmental triggers has been proposed to contribute to risk.

The clinical spectrum of PMR is wide but it classically manifests as bilateral aches and stiffness in the shoulders, neck and hips. Stiffness, a cardinal feature of PMR, is typically worse in the mornings and improves after periods of activity but may last all day [10]. These symptoms may cause difficulty elevating the shoulders, rising from a chair, turning over in bed or getting out of bed. The onset of symptoms may be over days, weeks or sometimes months, often accompanied by systemic symptoms such as malaise, fatigue, anorexia, weight loss and generalized arthralgia. Inflammatory markers (acute phase reactants including C-reactive protein, erythrocyte sedimentation rate and plasma viscosity) are usually elevated, but fever is less common [11]. Distal musculoskeletal features have been reported in 15–30% of people with PMR, including peripheral arthritis, distal swelling with pitting oedema and carpal tunnel syndrome [12]. Some patients initially diagnosed as PMR are later diagnosed with RA [13]. PMR can also be complicated by the development of GCA in ≈5–10% of cases [14] and a proportion of patients with PMR in secondary care cohorts may have GCA-like abnormalities on vascular imaging without any symptoms or signs of GCA [15]. In the absence of clinical features of GCA, the significance of such imaging findings is uncertain, particularly as atherosclerosis may also have a similar appearance on vascular imaging. Conversely, PMR-like symptoms are found in up to 50% of those diagnosed with GCA. PMR and GCA have some similarities, but they also have some differences.

The diagnosis of PMR is based on symptoms, signs and laboratory markers with a directed search for other conditions that can mimic PMR, based on the clinical presentation and context of care. Where there is diagnostic doubt that cannot be resolved clinically, advanced imaging may sometimes be used [16, 17], but to date, imaging tests for PMR are predominantly used for research purposes.

## Current practice

The aim of treatment is to relieve PMR symptoms and maintain symptomatic relief over time, while minimizing treatment side effects. Initial treatment is with glucocorticoids: typically, 15–25 mg prednisolone daily. In practice, 29–45% of patients with PMR do not respond completely to the initial treatment and ≥50% experience significant steroid side effects [3, 18]. The average duration of glucocorticoid therapy is usually quoted as between 1 and 2 years, but 25% of patients require >4 years of therapy [8, 19, 20]. The observed management heterogeneity of PMR arises from variations in clinical practice as well as the clinical heterogeneity of people with PMR.

It has been proposed that cumulative glucocorticoid burden might be reduced by administering glucocorticoids via periodic intramuscular or (peri)articular injection rather than via the oral route, but there are practical challenges to this as these injections must be delivered by a trained healthcare professional.

The disease course of PMR is complicated by relapses, with an estimated rate of 43% at 1 year [19]. Relapses are managed by increasing the glucocorticoid dose to the pre-relapse dose and tapering back down to the relapse dose over 4–8 weeks.

DMARDs are used successfully as a steroid-sparing agent with high efficacy in the management of many rheumatic conditions. Based on clinical trials data, the 2015 EULAR/ACR recommendations conditionally recommend early initiation of methotrexate, particularly in those at high risk of relapse and/or requiring prolonged glucocorticoid therapy [3]. However, there was no high-quality evidence to predict at the point of diagnosis which people with PMR were likely to relapse [3].

The evidence base for monitoring and follow-up for people with PMR is lacking. The current recommendations are consensus-based and guided by expert opinion. Some guidelines suggest that follow-up frequency could be as frequent as 1–4 weeks until disease remission [24], while other guidelines suggest every 1–4 months in the first year of diagnosis [2, 4]. The actual patterns of healthcare utilization of people diagnosed with PMR have been little studied, and these data are needed in order to plan better care pathways for this group.

Regarding future pharmacological therapies, the IL-6 pathway inhibitors tocilizumab and sarilumab have been studied in new-onset and refractory PMR [21–23]. Sarilumab has now been approved by the US Food and Drug Administration for treatment of people with PMR with an inadequate response to glucocorticoids or those who cannot tolerate a glucocorticoid taper. At the time of writing, neither IL-6 pathway inhibitor is approved in the UK or Europe for treatment of PMR. Other biologic DMARDs (bDMARDs) and targeted synthetic DMARDs (tsDMARDs) have also been evaluated in phase 2 trials and some phase 3 trials are now under way.

Non-pharmacological interventions such as physiotherapy, diet and nutritional supplements and complementary therapies have been little researched in PMR. The EULAR/ACR consensus recommends personalized exercise programs to maintain muscle mass and function and reduce the risk of falls, although acknowledging the limited evidence [3]. A UK cohort study of people with PMR found only 17% were offered physiotherapy, contrasting with other musculoskeletal conditions such as adhesive capsulitis and rotator cuff tears, which had referral rates >70% [25]. Physiotherapy is widely recognized as being useful for many musculoskeletal conditions. More research is needed to understand how non-medical health professionals can best add value in managing people living with PMR.

## Who the guideline is for

This guideline is for general practitioners, rheumatologists and general medicine physicians; specialist nurses and allied health professionals involved in the management of people with PMR; people with PMR and other stakeholders such as patient organizations.

There are no known equality considerations.

## What the guideline will and will not cover

The group that will be covered is people with PMR.

Areas that will not be covered include diagnosis of PMR [2], GCA [26] and immune checkpoint inhibitor–induced PMR [27].

Settings that will be covered include primary care and community settings and secondary and tertiary care settings.

## Activities, services or aspects of care

We will look at evidence in the following areas when developing the guideline, but it may not be possible to make recommendations in all the areas: pharmacological interventions; non-pharmacological interventions; management of relapses; follow-up and monitoring, including stopping treatment; outcome measures and goals for PMR treatment and patient information and support.

## Previous guidance

Previous guidance includes the 2015 recommendations for the management of polymyalgia rheumatica: a EULAR/ACR collaborative initiative [4] and the 2009 BSR and BHPR guidelines for the management of polymyalgia rheumatica [2].

## Key issues and draft questions

While writing this scope, we identified the following key issues and draft questions related to them. The key issues and draft questions will be framed in the Population, Intervention, Comparator, Outcome (PICO) format and used to develop more detailed review questions, which will guide the systematic review of the literature.

### Glucocorticoid dose

1. In people with PMR (P), what is the effect of the starting dose of glucocorticoids (I/C) on short-term remission of symptoms at 2–4 weeks (O)?
2. In people with PMR (P), what is the effect of the starting dose of glucocorticoids (I/C) on relapse risk, cumulative glucocorticoid dose, treatment-related adverse effects, quality of life and patient experience (O)?

### Glucocorticoid tapering

1. In people with PMR (P), what is the effect of the dose and interval of glucocorticoid tapering (I/C) on remission, relapse risk, cumulative glucocorticoid dose, treatment-related adverse effects, quality of life and patient experience (O)?
2. In people with PMR (P), what is the effect of prescribing a predefined glucocorticoid taper (I) on relapse risk, cumulative glucocorticoid dose, treatment-related adverse effects, quality of life and patient experience (O) compared with standard care (C)?
3. In people with PMR (P), what is the effect of a treat-to-target approach to treatment adjustments (I) on relapse risk, cumulative glucocorticoid dose, treatment-related adverse effects, quality of life and patient experience (O) compared with standard care (C)?
4. In people with PMR in clinical remission (P), what is the effect of the dose and interval of glucocorticoid tapering (I/C) on remission, relapse risk, cumulative glucocorticoid dose, treatment-related adverse effects, quality of life and patient experience (O)?

### DMARDs

1. In people with PMR (P), what is the effect of glucocorticoids combined with conventional synthetic DMARDs (csDMARDs) (I) on relapse risk, cumulative glucocorticoid dose, treatment-related adverse effects, quality of life and patient experience (O) compared with glucocorticoids alone (C)?
2. In people with PMR (P), what is the effect of glucocorticoids combined with bDMARDs or tsDMARDs (I) on relapse risk, cumulative glucocorticoid dose, treatment-related adverse effects, quality of life and patient experience (O) compared with glucocorticoids alone (C)?
3. In people with PMR (P), what is the effect of csDMARDs (I) on relapse risk, cumulative glucocorticoid dose, treatment-related adverse effects, quality of life and patient experience (O) compared with glucocorticoids alone (C)?
4. In people with PMR (P), what is the effect of bDMARDs or tsDMARDs (I) on relapse risk, cumulative glucocorticoid dose, treatment-related adverse effects, quality of life and patient experience (O) compared with glucocorticoids alone (C)?
5. In people with PMR (P), what is the effect of bDMARDs or tsDMARDs with or without glucocorticoids (I) on relapse risk, cumulative glucocorticoid dose, treatment-related adverse effects, quality of life and patient experience (O) compared with csDMARDs with or without glucocorticoids (C)?
6. In people with PMR (P), what is the effect of early introduction (within the first 6 months) of csDMARDs, bDMARDs or tsDMARDs (I) on relapse risk, cumulative glucocorticoid dose, treatment-related adverse effects, quality of life and patient experience (O) compared with delayed use (after 6 months) (C)?
7. In people with PMR who have relapsed (P), what is the effect of introduction of csDMARDs, bDMARDs or tsDMARDs (I) on relapse risk, cumulative glucocorticoid dose, treatment-related adverse effects, quality of life and patient experience (O) compared with glucocorticoid therapy alone (C)?
8. In people with PMR in clinical remission on csDMARDs, bDMARDs or tsDMARDs (P), with or without a stable maintenance dose of glucocorticoids, what is the effect (O) of tapering the DMARD dose (I) compared with not tapering the DMARD dose (C), while maintaining a constant dose of glucocorticoid therapy?

### Managing relapses

1. In people with PMR who relapsed on glucocorticoid tapering (P), what is the effect of the subsequent dose and interval of glucocorticoid tapering (I/C) on remission, relapse risk, cumulative glucocorticoid dose, treatment-related adverse effects, quality of life and patient experience (O)?
2. In people with PMR who have relapsed on glucocorticoid tapering (P), what is the effect of increasing the glucocorticoid dose to the pre-relapse dose (I) compared with adding a csDMARD, bDMARD or tsDMARD with or without glucocorticoids (C) on remission, relapse risk, cumulative glucocorticoid dose, treatment-related adverse effects, quality of life and patient experience (O)?

### Other management

1. In people with PMR (P), what is the effect on relapse risk, cumulative glucocorticoid dose and treatment-related adverse effects, quality of life and patient experience (O) of additional group or one-on-one care from non-medical healthcare professionals (nurse, physiotherapy, occupational therapy, psychologist) (I) compared with standard care (C)?
2. In people with PMR (P), what is the effect of providing written information on self-management (e.g. diet, physical activity) (I) on relapse risk, cumulative glucocorticoid dose, treatment-related adverse effects, quality of life and patient experience (O) compared with standard care (C)?

## Data Availability

No new data were generated or analysed in support of this research.
